# Scoping review of Neglected Tropical Disease Interventions and Health Promotion: A framework for successful NTD interventions as evidenced by the literature

**DOI:** 10.1371/journal.pntd.0009278

**Published:** 2021-07-06

**Authors:** Caroline Ackley, Mohamed Elsheikh, Shahaduz Zaman

**Affiliations:** 1 Global Health and Infection Department, Brighton and Sussex Medical School, University of Sussex, Brighton, United Kingdom; 2 Mycetoma Research Centre, Khartoum, Sudan; Kerman University of Medical Sciences, ISLAMIC REPUBLIC OF IRAN

## Abstract

**Background:**

Neglected Tropical Diseases (NTDs) affect more than one billion people globally. A Public Library of Science (PLOS) journal dedicated to NTDs lists almost forty NTDs, while the WHO prioritises twenty NTDs. A person can be affected by more than one disease at the same time from a range of infectious and non-infectious agents. Many of these diseases are preventable, and could be eliminated with various public health, health promotion and medical interventions. This scoping review aims to determine the extent of the body of literature on NTD interventions and health promotion activities, and to provide an overview of their focus while providing recommendations for best practice going forward. This scoping review includes both the identification of relevant articles through the snowball method and an electronic database using key search terms. A two-phased screening process was used to assess the relevance of studies identified in the search–an initial screening review followed by data characterization using the Critical Appraisal Skills Program (CASP). Studies were eligible for inclusion if they broadly described the characteristics, methods, and approaches of (1) NTD interventions and/or (2) community health promotion.

**Principal findings:**

90 articles met the CASP criteria partially or fully and then underwent a qualitative synthesis to be included in the review. 75 articles specifically focus on NTD interventions and approaches to their control, treatment, and elimination, while 15 focus specifically on health promotion and provide a grounding in health promotion theories and perspectives. 29 of the articles provided a global perspective to control, treatment, or elimination of NTDs through policy briefs or literature reviews. 19 of the articles focused on providing strategies for NTDs more generally while 12 addressed multiple NTDs or their interaction with other infectious diseases. Of the 20 NTDs categorized by the WHO and the expanded NTD list identified by PLOS NTDs, several NTDs did not appear in the database search on NTD interventions and health promotion, including yaws, fascioliasis, and chromoblastomycosis.

**Conclusions:**

Based on the literature we have identified the four core components of best practices including programmatic interventions, multi sectoral and multi-level interventions, adopting a social and ecological model and clearly defining ‘community.’ NTD interventions tend to centre on mass drug administration (MDA), particularly because NTDs were branded as such based on their being amenable to MDA. However, there remains a need for intervention approaches that also include multiple strategies that inform a larger multi-disease and multi-sectoral programme. Many NTD strategies include a focus on WASH and should also incorporate the social and ecological determinants of NTDs, suggesting a preventative and systems approach to health, not just a treatment-based approach. Developing strong communities and incorporating social rehabilitation at the sublocation level (e.g. hospital) could benefit several NTDs and infectious diseases through a multi-disease, multi-sectoral, and multi-lateral approach. Finally, it is important the ‘community’ is clearly defined in each intervention, and that community members are included in intervention activities and viewed as assets to interventions.

## Author summary

Neglected Tropical Diseases (NTDs) affect more than one billion people globally. A person can be affected by more than one disease at the same time. Many of these diseases are preventable, and could be eliminated with various public health, health promotion and medical interventions. This scoping review aims to determine the extent of the body of literature on NTD interventions and health promotion activities, and to provide an overview of their focus while providing recommendations for best practice going forward. Through a database search and by identifying appropriate literature 75 articles were identified that specifically focus on NTD interventions and approaches to their control, treatment, and elimination, while 15 focus specifically on health promotion and provide a grounding in health promotion theories and perspectives. Based on the literature we have identified the four core components of best practices including programmatic interventions, multi sectoral and multi-level interventions, adopting a social and ecological model and clearly defining ‘community.’

## Introduction

‘Neglected tropical diseases’ (NTDs) first entered the global health discourse following the creation of the United Nations’ Millennium Development Goals [1], whose aim was to combat poverty, hunger, disease, illiteracy, environmental degradation, and discrimination against women [2]. NTDs include a diverse group of communicable diseases that affect more than one billion people, with a further 1 billion at risk [3]. The majority of NTDs occur in the tropics and sub-tropics and cost economies billions of dollars every year [4].

The category of NTDs is socially constructed ‘as a result of certain diseases becoming an object of interest as a collective’ [1], yet the initial stimulus to grouping the ‘NTDs’ was their amenability to mass drug administration (MDA), and the support received to tackle them in the form of drug donations from key pharmaceutical patners. This justifies the early focus on MDAs and the relatively narrow list of NTDs adopted by some organisations, including the Bill & Melinda Gates Foundation. A Public Library of Science journal dedicated to NTDs lists almost forty NTDs [5], while the WHO prioritises twenty NTDs [4] and within that has identified ‘core’ NTDs, including those caused by parasitic worms (ascariasis, trichuriasis, schistosomiasis, lymphatic filariasis, onchocerciasis, dracunculiasis), bacterial infections (Buruli ulcer, leprosy, and trachoma), and protozoan infections (human African trypanosomiasis, Chagas disease, and Leishmaniasis) [6]. They are defined operationally on the basis of perceived commonality in their ‘spatial epidemiology and their mutual marginalisation in terms of research and funding’ [1]. They also have particular characteristics in common including: afflicting those living in poverty without access to safe water, sanitation, and basic health services; being chronic and slow developing conditions that progressively worsen if undetected and untreated; and whose damage can be irreversible; causing severe pain and life-long disability; causing stigmatization and social exclusion, leading to mental health issues [3].

A person can be affected by more than one disease at the same time from a range of infectious and non-infectious agents including, viruses, bacteria, protozoa, and helminth parasites [3]. Transmission is equally diverse and can take place via flies, fomites, fingers, snails, food, and the faeco-oral route (ibid.). For some NTDs like mycetoma, the causal agents (fungal or bacterial) and mode of transmission (thorn pricks) are still under research [7], while for others, like Dracunculus medinensis or "Guinea-worm, " the mode of transmission is more certain (‘drinking contaminated water from ponds or shallow open wells’) and understanding the complexities of the disease is better known (‘the cyclops is dissolved by the gastric acid of the stomach and the larvae are released and migrate through the intestinal well where after one hundred days the male and female meet and mate. The female moves down the muscle planes and after about one year of the infection the female worm emerges, usually from the feet, also releasing thousands of larvae and thus repeating the life cycle’) [8]. NTDs can lead to ‘blindness, deformity and disablement, disfigurement, cancer, and neurological problems’[3]. In addition to their impact on health, NTDs contribute to an immense social and economic burden resulting in social stigma, physical disability, and discrimination [9], as well as making it difficult to farm or earn a living, and limiting productivity in the workplace [10].

Many of these diseases are preventable, and could be eliminated with various public health, health promotion and medical interventions. In 2009, the WHO set out a human rights-based approach to health which included specific guidance for NTDs [11,12]. A human rights-based approach ‘requires that health interventions support the capacity of duty bearers’, usually governments, ‘to meet their obligations and of affected communities to claim their rights’ [11] The right to health is based on the principles of availability, accessibility, acceptability, and quality while addressing the underlying determinants of health and health care (ibid.). The WHO has set out a global plan to combat NTDs based on the principles of a right to health, using existing health systems for interventions, coordinating health system responses with other sectors, integration and equity, and a focus on control as part of ‘pro-poor policies’[12]. Within this the WHO is supporting country efforts to include NTDs in public health packages, allot budgets for de-worming programmes in schools, taking a multi-disease approach, and eliminating stigma and discrimination associated with NTDs through national campaigns (ibid.).

However, within this rights-based approach the biological diversity of NTDs means that control or elimination strategies need to be equally diverse. Some NTDs can be ‘controlled by drug treatment (preventative chemotherapy), on a country or community scale, via MDA. Other NTDs require different approaches and strategies for control or elimination, including specialised drugs and/or vector control (limiting or eradicating insects–e.g. flies and bugs–that transmit the pathogens)’ [3]. The WHO recommends five specific interventions for the control, elimination, and eradication of the NTDs: preventive chemotherapy by MDA; innovative and intensified disease management; vector ecology and management; veterinary public health services; and the provision of safe water, sanitation, and hygiene (WASH) [13]. The US takes an integrated control approach targeting multiple NTDs simultaneously through MDA, combined with community-level transmission control measures [14] Additional measures such as promoting clean WASH and good veterinary public health also play critical roles in addressing the underlying causes of NTDs (ibid.).

In this paper we aim to conduct a scoping review to determine the scope of the literature on interventions and health promotion activities on NTDs. We follow the International Classification of Health Interventions developed by the WHO where ‘a health intervention is an act performed for, with or on behalf of a person or population whose purpose is to assess, improve, maintain, promote or modify health, functioning or healthy conditions’ [4]. We include the five WHO recommended NTD interventions while also taking mapping, modelling, and health economics to be vital contributors to the control, elimination, and eradication of NTDs. As such they have been included in the review as preliminary activities, without which interventions would not be as effective.

This review was guided by the questions, ‘what are the approaches and methodologies used in NTD interventions?’ and ‘what characteristics and approaches are used in community health promotion theory that could be applied to NTD interventions?’. For the purposes of this study, a scoping review is defined as a research synthesis that aims to ‘map the literature on a particular topic or research area and provide an opportunity to identify key concepts; gaps in the research; and types and sources of evidence to inform practice, policymaking, and research’ [15]. We will identify best practice in NTD interventions and health promotion theory and suggest characteristics, approaches, and methodologies from both sets of literature that could be applied together to produce a framework for improved NTD interventions. We conclude the paper by suggesting a framework of best practice that combines successful approaches in NTD interventions and health promotion theory.

## Methods

This review includes both the identification of relevant articles through the snowball method and the methodological framework of Arksey and O’Malley [16].

### Data sources and search strategy

An initial search of the literature on NTD interventions and community health promotion theory using the snowball method [17] was implemented in March 2020 through a search of Google Scholar and the University of Sussex (UoS) library database with no date restrictions; only the first 100 hits (as sorted by relevance by Google Scholar and UoS) were screened. Reference lists were searched to identify further articles. In addition, a search of relevant course reading lists at UoS and the London School of Hygiene and Tropical Medicine was conducted. After a preliminary synthesis of the findings, a second search was implemented on 14 May 2020 in three electronic databases: PubMed, Academic Search Complete, and SciVerse Scopus (Elsevier). Articles from 2000 to 2020 were included in the Academic Search Complete and SciVerse Scopus database searches while full-text coverage was included in the MEDLINE data search, and only articles available in English were considered.

### Citation management

Citations were imported into the web-based bibliographic manager EndNote Clarivate Analytics, and duplicate citations were removed manually.

### Key search terms

Different keywords were used in relation to the different components of the study: NTD*, neglected tropical disease*, intervention*, strategies, best practice, community, and health promotion.

### Eligibility criteria

A two-phased screening process was used to assess the relevance of studies identified in the search. Studies were eligible for inclusion if they broadly described the characteristics, methods, and approaches of (1) NTD interventions and/or (2) community health promotion.

### Title and abstract relevance screening

The first level of screening reviewed the title and abstract of citations to immediately remove articles that did not meet the minimum inclusion criteria. Articles were screened by the authors based on the following criteria:

1. Does the article describe an NTD intervention?
2. Does the article describe any preliminary steps for effective NTD interventions, namely disease mapping and modelling or health economics?
3. Does the article describe characteristics and approaches of health promotion?
4. Is the article in English?

1,593 hits amongst the three databases underwent title and abstract relevance screening. Only the first 100 hits in SciVerse Scopus (as sorted by relevance by Scopus) and Academic Search Complete (as sorted by relevance by EBSCO) were screened after consideration of the time required to screen each and because it was believed that further screening was unlikely to yield additionally relevant articles [18]). If the article met either criteria 1 or 2, and 3 then it was included in further screening and appraisal. 74 articles were determined to meet the inclusion criteria from the database search. Using the snowball method 24 peer-reviewed journal articles, book chapters, websites, and news releases met the criteria. Duplicates were manually removed resulting in a total of 98 articles that met the inclusion criteria and were considered in the next phase of data characterization.

### Data characterization

After title and abstract screening all the citations deemed relevant were produced for subsequent review of the full text article. Relevant articles were then classified in a table with basic descriptive information [19]. Articles were appraised for appropriateness and rigor of research design and method, reliability, validity, and transparency of the study. The Critical Appraisal Skills Programs (CASP) was used by the authors to apply a three-point scale to each criterion (0 = criterion not met; 1/P = criterion partially met; 2/T = criterion met) [20]; Critical Appraisal Skills Program 2020). Ninety articles met the CASP criteria partially or fully and then underwent a qualitative synthesis.

### Data summary and synthesis

The articles underwent a qualitative synthesis and were compiled in a single spreadsheet on Microsoft Excel. The following information was recorded for each article:

1. Title and author(s)
2. Journal and database/search method
3. DOI and year published
4. Time and geographical location(s)
5. NTD (as appropriate)
6. Type of intervention/preliminary steps to intervention and duration
7. Research design and methods
8. Outcome indicator
9. Important results

Qualitative methods (interviews and/or focus group discussions) and/or cross-sectional surveys were appropriately deployed in studies exploring: the knowledge, attitude, and practice of identified populations; participants’ perspectives and experiences of NTD service provision and uptake from a gendered perspective; level of care in health systems; community engagement interventions; behaviour change and health promotion interventions; experiences of MDA; and WASH prevention strategies.

The methods of diagnostic testing (e.g. through mathematical modelling) and disease mapping and modelling (e.g. precision mapping, integrated mapping) included appropriate outcome indicators (e.g. detection of new cases) in their study design, with some which test the hypothetical effectiveness of methods (e.g. agent based modelling) proving appropriate for this scoping review as it includes both disease agent and human decision analyses.

Interventions that used methods to explore cost-effectiveness ranged from MDA, complimentary health interventions (e.g. simultaneous WASH, de-worming, and school feeding programs), and regional collaboration in NTD elimination programs. The methods used were appropriately deployed and it was determined that by analysing the outcome indicators at a conceptual level (e.g. ‘trade-offs between the different option’s costs and effects’ [21] proved most useful in determining appropriateness for this scoping review.

Ten articles identified in the search strategy did not deploy a specific intervention or undertake a research study, however, they illuminate important results and address the research questions that guide this scoping review. These include policy briefs (1) (e.g. analysis of African health systems for the diagnosis and management of chronic diseases, including NTDs), literature reviews (5) (e.g. review of the literature on WASH interventions and NTDs), data summaries (3) (e.g. summary of challenges to introduce new technologies that will eliminate NTDs; summaries of economic control measures in NTD interventions), and public statements (1) (e.g. calling for capacity building for local scientists). Six studies identified in the search strategy were initially included in the data summary and removed before data analysis as they did not address the research questions due to inappropriate or irrelevant study design (e.g. does not address NTDs but addresses health promotion), sample populations (e.g. bovine population), interventions (e.g. protein identification), or research methods (e.g. study protocol).

### Data analysis

**Fig 1:**
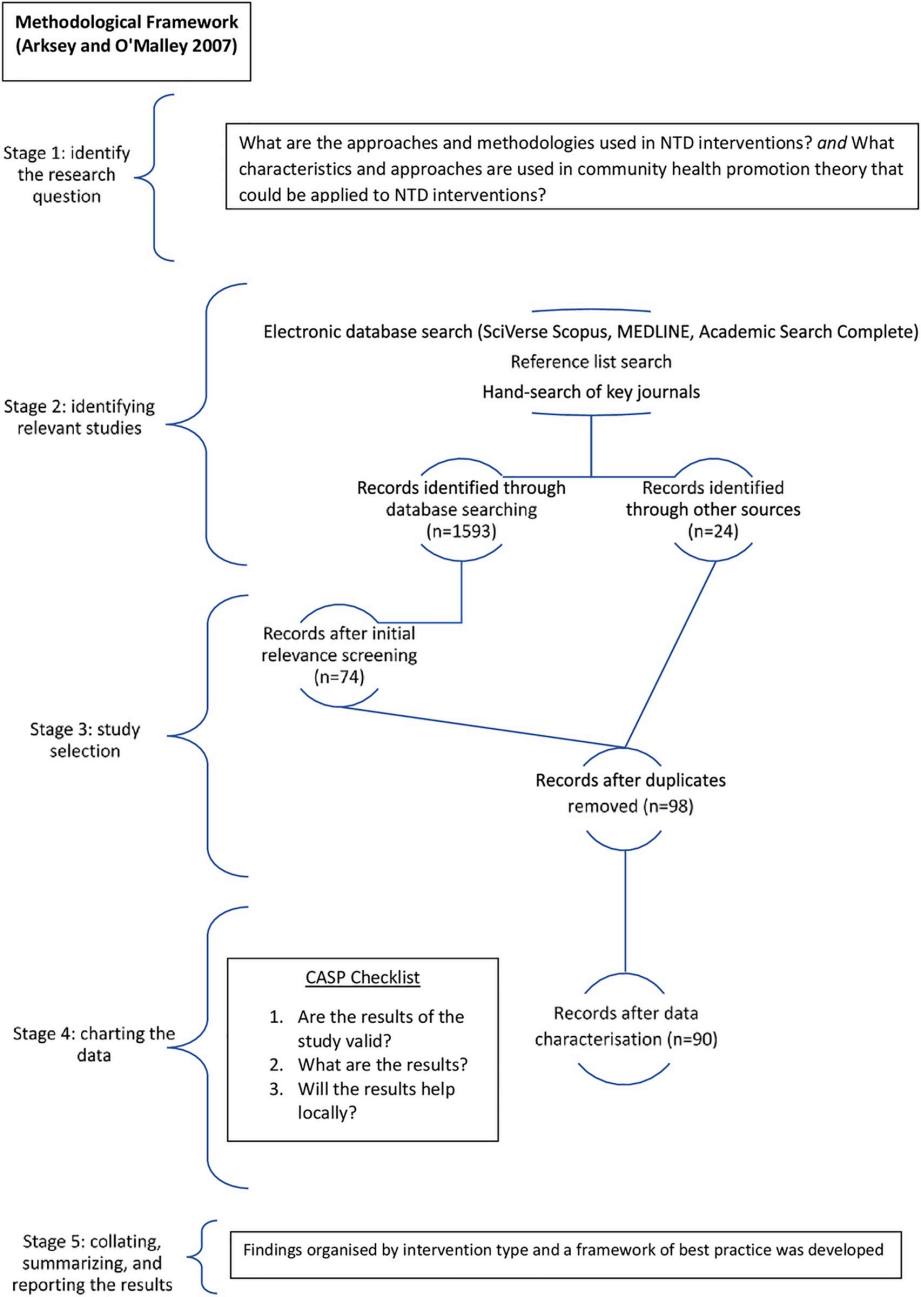
Flowchart diagram of total number of articles identified and selected.

Articles were analysed using narrative analysis to describe, summarise, and synthesise the findings. Descriptive summaries of each paper were reviewed to identify codes according to common themes across papers that answer the research questions. The analysis also compares studies [19,22] according to intervention type. Basic characteristics of the studies were summarised and interventions or their preliminary steps were analysed according to sample size, participants, economic aspects, methods, outcomes, effectiveness, and gaps in the research [16]. This consistent approach to reporting findings allowed for comparisons to be made across intervention types while reducing potential bias (ibid). The following figure (Fig 1) shows the flowchart diagram of total number of articles identified and selected.

## Results

### NTDs covered and their locations

Of the ninety articles reviewed, seventy-five specifically focused on NTD interventions or their preliminary steps and approaches to their control, treatment, and elimination, while fifteen focused specifically on health promotion and provide a grounding in health promotion theories and perspectives. A majority (29) of the articles on NTDs provided a global perspective to control, treatment, or elimination through policy briefs or literature reviews. Twenty-seven of the articles focused on NTD elimination in Africa with one taking a broader continental perspective and two focused specifically on sub-Saharan Africa. A majority of the articles on NTDs in Africa were based on specific case studies in East and Southern Africa with two taking a multi-country approach. Thirteen articles identified research on NTDs in Asia with a regional focus on Southeast Asia and the Western Pacific.

Of the seventy-five articles on NTD interventions or preliminary steps and approaches to their control, treatment, and elimination a majority (19) of research focused on providing strategies for all NTDs while twelve addressed multiple NTDs (e.g. country specific clusters in Togo—schistosomiasis, soil transmitted helminthiases, and trachoma; or in Mali and Senegal- lymphatic filariasis, trachoma, schistosomiasis and soil-transmitted helminths) or their interaction with other infectious diseases (e.g. HIV/AIDS, TB, and malaria) for a comprehensive approach. Four classified their studies according to specific interventions (e.g. MDA including deworming, vitamin supplements, and child immunization; use of smart phones by community health workers; and national school health programmes). Two studies focused on contextual factors like concerns around WASH or route of transmission like NTDs where infection is predominately through the feet or soil transmitted helminthiases. The remaining thirty-six articles focused on specific NTDs, with a majority of those on schistosomiasis (6), followed by dengue (4) leprosy (3), soil transmitted helminths (2), podoconiosis (2), lymphatic filariasis (2), leishmaniasis (2), human African trypanosomiasis (2), onchocerciasis (1), trachoma (1), hantaviruses (1), and Buruli ulcer (1). Two articles addressed NTDs that the authors felt needed greater recognition, one calling for infectious corneal ulceration to be identified as an NTD and another addressing enteric pathogens (Shigella, Salmonella, E. coli) which are currently listed by PLOS NTDs as NTDs, but not by the WHO.

Of the twenty NTDs categorized by the WHO and the expanded NTD list identified by PLOS NTDs, several NTDs did not appear in the database search on NTD interventions and/or health promotion, including yaws, fascioliasis, and chromoblastomycosis[23]. Table 1 shows the NTDs covered and their locations.

**Table 1:** NTDs covered and their location.

NTDs covered	Location(s)
All NTDs	Global; Africa; Sub-Saharan Africa; Ethiopia; Uganda; Tanzania; Southern Sudan; Mozambique
NTDs requiring MDA	Uganda
NTDs attributed to water, sanitation, and hygiene (WASH)	Tanzania
NTDs for which the route of transmission or occurrence may be through the feet (cutaneous larva migrans (CLM), leptospirosis, mycetoma, myiasis, podoconiosis, snakebite, tungiasis, and soil-transmitted helminth (STH) infections)	Global
Enteric pathogens (Shigella, Salmonella, E. coli) *Identified as an NTD by PLOS One NTDs, *but not the WHO*	Laos
Buruli ulcer	Benin
Chagas disease	Global; Bolivia; Ecuador
Dengue	Global; Philippines; China; Malaysia; Myanmar
Human African Trypanosomiasis	Global; South Sudan; Democratic Republic of Congo
Helminth NTDs (including soil transmitted helminths and intestinal helminthiases)	Global; Southeast Asia; Western Pacific Region; Zambia; Kenya; Philippines; Togo; Madagascar; Mali; Senegal; Ethiopia
Leishmaniasis (including Visceral Leishmaniasis (VL) and American Cutaneous Leishmaniasis (ACL))	Global; Colombia; India; Brazil
Leprosy	Global; China; India; Colombia
Lymphatic Filariasis	Global; Southeast Asia; Nepal; Zambia; Madagascar; Mali; Senegal; Ethiopia; Kenya
Onchocerciasis	Global; Cameroon
Podoconiosis	Ethiopia
Rabies	Tanzania
Schistosomiasis	Global; Southeast Asia; Sub-Saharan Africa; Ethiopia; Kenya; Zanzibar; Togo; Tanzania; Madagascar; Mali; Senegal
Trachoma	Global; Southeast Asia; Togo; Mali; Senegal; Ethiopia

### Global approaches and policy briefings on NTD control, treatment, and elimination

The WHO and the US government have developed guidelines that many NTD interventions have adopted. The WHO recommends five interventions for the control, elimination, and eradication of the NTDs: preventive chemotherapy by MDA; innovative and intensified disease management; vector ecology and management; veterinary public health services; and the provision of safe WASH[23]. Recently, the WHO NTD Department has highlighted the need for a cross-sectorial, cross-disease approach that is laid out in the recently released Roadmap 2021–2020 [4].

The US recommends an integrated control approach targeting multiple NTDs simultaneously through MDA, combined with community-level transmission control measures[14]. Additional measures such as promoting clean WASH and good veterinary public health also play critical roles in addressing the underlying causes of NTDs (ibid.). The CDC [10] conducted a review of global NTD interventions illuminating an emphasis on MDA and WASH related activities, including the following disease specific measures:

- Lymphatic Filariasis: provision of anti-parasitic drugs, hygiene education (helped reduce stigma), access to surgery
- Onchocerciasis Control: mass drug administration, surveillance
- Schistosomiasis: provision of praziquantel
- Soil-transmitted Helminths: drug provision and WASH education aimed at prevention
- Trachoma: surgery, antibiotics, facial cleanliness, environmental educational efforts
- Guinea Worm: improving unsafe water

What becomes evident from these leading bodies is that a multifaceted approach that targets multiple diseases through clinical intervention with medication provisions and a focus on social and environmental determinants across sectors, namely WASH education, is vital. It also became apparent that country guidelines are not often enough made accessible through standard database searches, perhaps limiting cross-country knowledge exchange.

### Preliminary steps to effective interventions

For NTD interventions to be effective preliminary research is necessary. Disease mapping, vector modelling, and cost benefit analysis are all important to consider when designing and implementing interventions and evaluating their potential impact. In this section, we describe the literature identified in the database search on diagnostic screening and disease surveillance, mapping and modelling of NTDs, and health economics.

### An emphasis on diagnostic screening and disease surveillance

In 2012 the WHO raised hope that many NTDs can be eliminated by devising guidance in the form of a road map which has new been revised with updated strategies for the period of 2021–2030. The road map includes key measures to be implemented, including surveillance tools and response approaches. They note that effective surveillance and risk-mapping that visualises infection trends through geospatial approaches improves the efficiency of control measures as well as decision making at the technical and political level. They also indicate that detection and interruption of transmission requires a focus on diagnosis as part of integrated public health response packages that are tailored for the context; thus, surveillance is both an intervention approach and an early warning system for (re)emergence of endemic infections and for new epidemics and pandemics [24].

There is a renewed emphasis on measures for early detection of leprosy in post-elimination era Shandong province, China.[25] note that there were few new leprosy cases in Shandong and they were detected by ‘passive modes’ where ‘advanced cases’ and ‘visible disability were common’. The authors reviewed cases detected during 2007–2017 and conclude that although there are few cases, a leprosy control program is still needed. They suggest such a program ought to include government financial support and policy as well as comprehensive case-finding measures in former high and middle endemic areas. They argue that symptom surveillance is vital for early detection of new cases, along with health promotion, personnel training, and reward offering.

To achieve the goals of elimination and zero transmission of Gambian human African trypanosomiasis (HAT) by 2020 and 2030 respectively, current interventions may not be sufficient according to [26]. They developed a mathematical model of disease dynamics to assess the impact of changing intervention strategies in two high endemic areas of the DRC. They found that effectiveness of HAT screening needed to be improved by recruiting high-risk groups while also identifying seven vector control strategies needed to reduce transmission. They recommend a two-pronged strategy including enhanced active screening and tsetse vector control to ensure the success of the control program in the DRC and to meet the 2030 goal of elimination [27] undertook a literature review to assess the diagnostics and control of neglected tropical helminth diseases. They found that progress has been made but note that additional actions are needed to maintain the achievements of investments in NTD control. The scaling up of MDA through control interventions has led to a reduction in soil transmitted helminths. They note that improved hygiene, with a focus on endemic areas, has led to a considerable reduction in the incidence of lymphatic filariasis (LF). Yet, they suggest that identifying communities with potential cases remains a challenge for African programme managers and that public-private partnerships are key to the management of NTDs.

### Importance of mapping and modelling

Effective identification, control, treatment, and elimination of NTDs requires disease mapping and modelling to correctly identify endemic areas and disease prevalence. In a review of NTDs in Ethiopia, [28] identify that Ethiopia bears a significant burden of NTDs compared to other sub-Saharan African countries. The authors undertook a review to inform an Ethiopian NTD Master Plan and found that to achieve success in integrated control of NTDs integrated mapping along with the rapid scale up of interventions and research into implementation is vital.

Several interventions identified in the database search emphasised innovative mapping and modelling techniques. [29] field tested an integrated mapping protocol for NTDs in Mali and Senegal through a methodology called integrated threshold mapping (ITM). They argue that where geographic overlap among NTDs exist an integrated mapping approach could result in better cost effectiveness of interventions, and they found that ITM was practical, feasible and demonstrated cost savings when compared with the standard non-integrated WHO methodologies.Tchuem Tchuente et al [30] Tchuem Tchuente et al [29]focus on precision mapping, or sampling at a ‘finer’ geographic resolution, of schistosomiasis in Cameroon to address limitations in the current conventional mapping design. They suggest that uncertainties and misclassification of endemic locations risks successful preventative chemotherapy coverage for all populations that need treatment. In contrast to a focus on mapping to administer effective treatment for disease elimination Miksch, Jahn et al [31] write about agent-based models (ABM) to allow for dynamic transmission modelling. ABM modelling can reproduce direct and indirect effects of interventions as well as replicate human behaviour. The outcomes of this type of modelling include prevalence and incidence of infected persons, which is vital in planning treatment, control, and elimination. In a study of dengue interventions in the Philippines ABM identified that a reduced human to mosquito ratio during the rainy season led to a substantial decrease in infected persons.

### Cost benefit analysis for effective interventions

Liese and Schubert [32] analysed official development assistance (ODA) commitments for infectious disease control from 2003 to 2007 to understand how neglected NTDs really are. They found that development assistance committee (DAC) countries and multilateral donors have largely overlooked funding NTD control projects. Only 0.6% of total annual health ODA was dedicated to the fight against NTDs and does not reflect the health burden of NTDs. Horton, Gelband et al [33] reviewed 93 health interventions for major infectious diseases (AIDS, TB, malaria, and NTDs) in LMICs from 2005–2006 and ranked them by cost-effectiveness. They argue that cost-effectiveness rankings of health interventions are useful for national healthcare planning and budgeting. They also acknowledge that since 2006 many priorities have changed due to new technologies, new methods for changing behaviour, and price changes for vaccinations and treatment drugs. Yet, they suggest that regularly updating cost-effectiveness rankings on a regular basis is valuable for achieving the Sustainable Development Goals.

Lee, Bartsch et al[21] conducted an economic and financial evaluation of NTDs. Studies on the economics and financing of interventions are important for decision making when resources are scarce or limited, as is the case for NTDs. They suggest that one reason there is a shortage of resources for NTD control is because the burden of NTDs and the economic value of control measures may have not been fully characterised. They argue that economic studies can help various decision makers–funder, researcher, manufacturer, employer, policy maker, and health care worker–see each other’s points of view and bring people together. Hotez and Ehrenberg [34] examined ways to escalate NTD action through a global network for NTDs that implemented a finance mechanism to control the most common NTDs across 11 Southeast Asian countries, including China. They advocate for regional plans of action, endorsed by ministries of health, for NTD prevention, control, and elimination. They suggest that such plans will provide the framework for national plans and resource mobilization to support regional fundraising to combat NTDs.

The database search revealed several case study examples of studies that focused on cost-effectiveness and impact of targeted interventions. De Neve, Andriantavison et al[35] did extended cost-effectiveness analysis for five NTDs in Madagascar using methods to assess the health gains, household financial gains, and education gains of preventative chemotherapy. They found that by incorporating both the financial and educational gains in the economic evaluation of health interventions additional information about attainment of the Sustainable Development Goals became available. Hounsome, Kassahun et al[36] explored the cost-effectiveness and social outcomes of a hygiene and foot-care intervention for people with podoconiosis in Ethiopia. They found that this community-based intervention, which included training in foot hygiene, skin care, bandaging, exercises, and use of socks and shoes benefitted the entire community with the greatest impact for the poorest.

### Level of intervention and care

The level at which interventions ought to be implemented differs greatly across disciplinary approach, disease, country, and funding body. Findings of a cross-sectional survey on *Schistosoma mansoni*, soil-transmitted helminths, *Taenia* species, and *Entamoeba histolytica/dispar* in rural Kenya by de Glanville, Thomas et al[37] suggest that social and environmental contextual conditions shape infection at the household, sublocation, and constituency levels and may provide actionable targets for public health interventions to reduce both the prevalence of infection as well as health inequality. They argue that NTDs are characterized by their tendency to cluster within groups of people, yet the measures of clustering are rarely reported in community-based studies of NTD risk. There is evidence suggesting that a multi-sectoral and multi-disease approach to NTDs has considerably more impact than relying on the health sector alone.

Nakagawa, Ehrenberg et al[38] argue that the health sector cannot address the multi-faceted determinants of NTDs. They write that the control and eventual elimination of helminth NTDs will require moving beyond preventative chemotherapy to addressing the root social and ecological causes of transmission. They identify the following environmental determinants to be associated with helminth NTDs: water supply, sanitation, school canteens, and classroom overcrowding. According to the researchers a lack of access to clean water and adequate sanitation, coupled with poor hygiene and other determinants associated with low socioeconomic development often allow infection rates to return to baseline levels within a year of drug administration.

Weng, Chen et al [39] conducted a review of the literature and found that the implementation of multilateral collaborations leads to continued efforts and plays a critical role in drug discovery. Proactive approaches and advanced technologies are needed in NTD drug innovation. They also suggest that since NTDs are closely linked to poverty, it is essential that stakeholders take measures to alleviate poverty, strengthen social interventions, adapt to climate change, provide effective monitoring and ensure timely delivery of interventions.

Olveda, Leonardo et al[40] take a regional focus in their level of intervention to tackle multiple NTDs together through analysis of the Regional Network on Asian Schistosomiasis and Other Helminth Zoonoses (RNAS+). They state that there is now general agreement that control activities need research collaboration to progress, while surveillance plays an increasingly important role in sustaining long-term relief. In addition, the RNAS+ has found that a focus on not only strengthening research capabilities but also on furthering efforts to close the gap between research and control and bridge different branches of science are vital. They illustrate that regional bodies like RNAS+ provide a more rapid translation of research results into control applications and disseminate data and new technology through networking. Such an approach improves the overall situation of multiple NTDs in a large geographic area.

Holistic care is at the focus of a qualitative study by Jung, Han et al[41] that explores the in-depth contextual attributes of an Indian hospital community that has been able to successfully provide sustainable leprosy care without excessive external funds. The findings of their qualitative study indicate the importance of forming a cooperative community and implementing social rehabilitation for leprosy control. They argue that social sustainability is only possible through holistic care, which includes psychosocial, educational, medical, and residential support.

Interventions need to consider the flow of information to ensure that outputs include several levels of engagement for holistic care. Madon, Amaguru et al [42] conducted a qualitative case study design using mobile phones for Management Information System (MIS) at the village level in Tanzania. Their study looks at the flow of information when capturing data at the source (individual) to improve health systems at the district and national levels. They found that even with improved technology and political will, the biggest barrier to local usage of information for health planning is the lack of synthesised and analysed health information from the district and national levels to the villages. They argue that without instilling a culture of providing health information feedback to frontline workers and community organisations, the benefits of a MIS intervention will be limited. If not addressed, they suggest that mobiles will have maintained a one-way upward flow of information for NTD control and simply made reporting more hi-tech rather than effective in changing health systems.

Holistic care can focus not just on the hospital or individual level, but also at a systems level through an emphasis on health systems. Osakunor, Sengeh et al [43] take a health systems approach to promote effective and sustainable healthcare in Africa. They hypothesise that the increasing burden of non-infectious diseases may be correlated directly and indirectly to, or further exacerbated by, the existence of NTDs and other infectious diseases within a population. Some health systems have adapted to include management of chronic conditions, but there remains the challenge to equip health systems to shift from episodic interventions for acute care to be resourced for chronic care and to make existing support groups and systems available to the poorest and most vulnerable groups of affected patients. They suggest this can be achieved through health, research, and development capacity building of the next generation of scientists and health workers. Additionally, guidelines, drugs, and suitable monitoring equipment need to be in place, accessible and affordable to all and tailored to different levels of care. Finally, they highlight the importance of education through interactive media and mobile devices.

Decentralization of care is the focus of a study by Amoussouhoui, Sopoh et al [44] Buruli ulcer (BU) in Benin. In Benin, BU patient care is being integrated into the government health system, however the authors piloted a program designed to introduce decentralization in one of the country’s most endemic districts previously served by centralized hospital-based care. They found that early BU lesions (71% of all detected cases) could be treated in the community following outreach education, and that most of the afflicted were willing to accept decentralized treatment. They argue that outreach and a decentralized care program can successfully reach early BU cases and integrate wound management for NTD control in patients not previously treated by a proactive centralized BU program.

Nakagawa, Ehrenberg et al[38] reviewed the main determinants for helminth NTD endemicity and current control strategies in the Western Pacific. They identified several gaps in current programs that they suggest should be addressed for effective interventions. These suggestions include many of the recommendations in the above mentioned literature that span several levels of NTD intervention and care—(1) developing strategies to mitigate negative impacts on helminth NTD control, (2) identifying sectors best placed to carry out additional activities required to achieve helminth NTD control, (3) developing an integrated work plan (4) having a clear delineation of roles and responsibilities among actors, (5) building strong partnerships and collaborations between sectors, (6) ensuring continuous communication and exchange.

### Prevention

The NTD movement started on the basis that large amounts of drugs were freely donated for prevention of the ‘preventive chemotherapy’ (PC) NTDs—Lymphatic Filariasis (erroneously referred to as ‘elephantiasis’), Onchocerciasis (River Blindness), Schistosomiasis (Bilharzia), Trachoma, and Soil—Transmitted Helminthiasis (Intestinal Worms). Prevention has been central to the NTD elimination approach and treatment of people already affected has lagged behind. Building on this emphasis of prevention Fitzpatrick, Nwankwo et al [45] suggest that focusing on investing in medicines and universal healthcare will eliminate NTDs in the most cost-effective way. Similarly, Engels and Zhou [46] indicate that future challenges in elimination include the mainstreaming of NTD interventions into Universal Health Coverage and the coordination with other sectors to get to the roots of poverty and scale up transmission-breaking interventions. Ortu and Williams [47] argue that strengthening the capacity of the primary health care system will enable correct diagnosis and improved management of NTDs. They consider the primary health care system to be at the frontline of disease surveillance and that strengthening systems along with shifting financial support from disease-oriented programs to disease integrated interventions will have an impact on NTD elimination. In a slightly different approach Njenga, Mwandawiro et al [48] focus on target populations to prevent spread of NTDs to ‘at risk’ and more vulnerable groups. They argue that focusing on adult populations may contribute to a decrease in community transmission and lead to lower morbidities in children. This preventative approach is particularly impactful in its inclusion of systems (e.g. universal health care) that acknowledge and address social determinants of health, rather than a focus on behaviour change models (e.g. WASH education) that might increase health inequality [49].

### Interventions through mass drug administration

MDA is recommended in the treatment and elimination of NTDs. It is both a preventative and a reactionary intervention that can be applied in a range of ways. For example, the WHO [50] published a global update on the implementation of preventive chemotherapy (PC) against a group of helminthic diseases: lymphatic filariasis, onchocerciasis, schistosomiasis, soil-transmitted helminthiasis. They argue PC remains vital for control and elimination strategies, but that it must be coupled with other interventions to be successful; these interventions include morbidity management, vector control and access to safe WASH practices.

Odhiambo, Musuva et al [51] suggest that MDA be integrated with other health interventions after conducting a longitudinal qualitative study to explore the experiences, opportunities, challenges and recommendations of community health workers (CHWs) after annual MDA activities for schistosomiasis using the community directed intervention (CDI) strategy in Kisumu City, Kenya. They also recommend the CDI approach in MDA implementation based upon the presence of CHWs and their supervisory structures. Chami, Kabatereine et al [52] explored the best combination of community medicine distributors (CMDs) for MDA in Uganda. They found that to improve the effectiveness of CMDs, national programmes should explore interventions that facilitate communication, friendship, and equal partnership between CMDs.

Dorkenoo, Bronzan et al [53] suggest that prevalence survey mapping needs to be conducted to effectively implement MDA in Togo. This mirrors WHO guidelines that state mapping must precede requests for MDA and hence its distribution. They suggest that the results of mapping be used by ministries of health and partners to plan integrated MDA. In a call for increased information Toor, Turner et al [54] suggest treatment strategies ought to include more accurate and detailed data from monitoring and evaluation (M&E) programmes to accurately determine the best treatment strategy for a region. They reviewed previous schistosomiasis M&E programmes and found that prevalence and intensity of infection data from a larger age-range and better modelling into longitudinal adherence to treatment was needed to generate recommendations of PC levels needed.

### Interventions that focus on knowledge, attitudes, and practice

Understanding the knowledge, attitudes, and practices (KAP) of a community is important to improve the prevention and control of NTDs. Harris and Armién [55] explored hantavirus prevention practices as well as knowledge and attitudes based on the health belief model that includes perceived severity, susceptibility, obstacles, benefits, and cues to action in Panama. They note that prevention measures by community members are key to stopping outbreaks as most knowledge was learned through fellow community members, particularly those who have experienced the disease. Community awareness is essential to preventing disease spread, as noted by AhbiRami and Zuharah [56] in their study to assess the KAP regarding dengue among school children from flooded and unflooded areas in Malaysia. They found that health education programs for all age groups ensures the communication of positive knowledge and attitude changes. However, unlike in Panama, participants received most of their information through television, newspapers, and schools indicating the need for knowledge dissemination through a range of mechanisms. When introducing new control and treatment strategies, KAP can be useful to understand gaps in community knowledge and preferred channels for information dissemination.

In Yei County, South Sudan, Bukachi, Mumbo et al [57] found that education, gender, geographic location, and history of HAT all contributed to levels of knowledge of, and perceptions of the treatability of HAT. Additionally, most people got their information on HAT from the radio. The authors suggest that gaps in knowledge could be bridged through effective education and community strategies for HAT alongside other interventions.

### Community driven interventions

Many NTD programmes rely on community-driven approaches to maximise intervention access and performance. Community involvement can take many shapes, but it is a vital ingredient for sustained interventions. Das, Salam et al [58] suggest that community-based interventions ought to include the community in all aspects of disease eradication, from preventative measures (education campaigns and spraying of insecticide) to treatment (mass drug administration). A community approach can include the most basic of house-to-house screenings [59] or therapy management by community volunteers, particularly women [60]

Bardosh (2018) makes the case for a socio-anthropological framework with an emphasis on community knowledge to improve the effectiveness of NTD interventions. They conducted three rapid ethnographic studies on rabies elimination in Tanzania, sleeping sickness control in Uganda, and the prevention of parasitic worms in Zambia. The framework includes five ‘intervention domains’ where the ‘effectiveness of the interventions was negotiated and determined at the local level, including (1) the terrain of intervention (including seasonality and geographical variability); (2) community agency (including local knowledge, risk perceptions, behaviors, leadership and social pressure); (3) the strategies and incentives of field staff (skills, motivations, capabilities and support); (4) the socio-materiality of technology (characteristics of intervention tools and the adoption process itself); and (5) the governance of interventions (policy narratives, available expertise, bureaucracy, politics and the utilization of knowledge)’ (ibid). Bardosh places strong emphasis on the role of ‘field staff’ for improved efficacy and effectiveness of NTD programs. They write that field staff should contribute to knowledge generation about contextual factors, apply knowledge to tailored field strategies, and to support in the creation of mechanisms that facilitate implementation.

In another qualitative study Khan, Pullan et al [61] explored how ‘domestic stakeholders’ working in health, education, community engagement and sanitation perceived investments in different strategies for NTD control, particularly soil transmitted helminths (STH) in Kenya. Stakeholders criticised MDA for failing to address the underlying causes of STH and identified three priority areas for programme investment, (1) changes in institutions to reduce working independently of each other, (2) developing community demand and ownership, and (3) increased policymaker participation in the underlying socioeconomic and environmental causes of STH. The authors identify a need to shift responsibility for NTD programmes from external agencies to domestic stakeholders with a focus.

Madon, Malecela et al [62] took a mixed methods approach to understanding community participation in NTD and WASH intervention programmes in Tanzania. The authors note that in Tanzania the strategies aimed at reducing NTDs attributed to WASH problems tend to be top-down in nature. Alternatively, they suggest strengthening the governance of such programs by integrating vertical disease programs and by improving the efficiency of report-generation. They argue for community participation using existing village governance structures is an effective strategy for developing sustainable village health governance.

Although the literature identifies the important roles the community can take in NTD prevention and interventions, ‘community’ needs to be more clearly defined. It is helpful to define and clarify what ‘community’ is in any health promotion programme so interventions can be more effective. McLeroy, Norton et al [63] identify four main categories of ‘community-based’ in health promotion: (1) community as setting, (2) community as target, (3) community as agent, and (4) community as resource. ‘Community as setting’ is when community is defined geographically as the location where interventions will be implemented. The scale of community as setting can vary, including city-wide, neighbourhood, or institution (e.g. school) level. The primary focus in interventions that define community as a setting is to change individual behaviour to reduce population level risk of a disease. When ‘community’ is the target the goals is to create healthy community environments through broad changes in public policy, community institutions, and services. The desired outcome is a change in health. When the ‘community’ is the agent, they provide resources for meeting daily needs through structures like the family unit, social networks, businesses, or political structures. This is the least utilised model of community in public health, but they are naturally occurring units of solution that do not require professional intervention and supports community capacity. It is important to consider who might be excluded in this model, and to assess community structures and processes beforehand.

The fourth model is when ‘community’ is a resource, the most applied model in community health promotion. This model involves a high degree of community ownership and participants with outcomes achieved when actors work through a vast array of community institutions and resources. Unlike ‘community as agent,’ this model requires external resources to not only change individual behaviour, but also community capacity. This model also calls for a social ecology approach where the behaviour of individuals is in the social context. It should also be acknowledged that there is a role for the community to help develop interventions that are both effective and meaningful through a co-production approach. For example, the literature suggests that complementary and preventative WASH activities should accompany NTD mass drug administration, but the literature fails to illuminate the role the community played in developing and administering these activities. If communities are involved in the process and implementation of interventions then they will better align with individual needs and priorities, thus making them more sustainable and impactful.

### Behaviour change interventions

Behaviour change interventions are a common tool in global health, and the methods and approaches to engage people in such interventions vastly differ. Such interventions are both community driven and preventative in nature. For example, Dodson, Heggen et al [64] focused their intervention on facial cleanliness and environmental improvement programming. They suggest that behaviour interventions should be in partnership with communities and local actors to support three key decisions (1) what processes ensure effective, contextualized and participatory programme design? (2) what types of intervention should be developed? (3) how should specific programme components be selected and labelled for study and discussion? Bates, Villacís et al [65] attempted to understand which antecedents to behaviour changes are best to emphasise when promoting prevention of home infestation of Chagas disease in Ecuador through the Health Belief Model (HBM). However, they found that the HBM did not predict actual infestation, rather it could predict intentions to prevent. They note that it is necessary to assess home practices and their actual efficacy to develop messages to reduce home infestation by triatomine bugs. Drawing on a technological approach to behaviour change Luz, Masoodian et al [66] introduced a serious game for healthcare in Acre-Brazil to train people and to change attitudes and behaviours on Visceral Leishmaniasis (VL) and American Cutaneous Leishmaniasis (ACL). The game was founded on a process of collaboration that included computer scientists, designers, medical researchers, and practitioners.

### Health promotion and education interventions

Many behaviour change interventions incorporate aspects of health promotion and education and are preventative. Yet, the aim of how to promote health through education differs from the aim to change an individual’s or community’s behaviour. Several of the articles identified in the literature search focus on health promotion for participation in MDA while identifying the importance of community and school based delivery modes [67–69]. Schools and teachers can play a vital role in health promotion as evidenced by Celone, Person et al [70] who look at how to increase the reach of programmes by involving local Muslim religious teachers in Zanzibar to tackle urogenital schistosomiasis. They found that religious teachers valued the opportunity to educate students and were influential and effective change agents. They helped expand and increased acceptance of elimination activities while also increasing participation in such activities. In contrast to a focus on schools, Nieto-Sanchez, Bates et al [71] look at the Healthy Homes for Health Living (HHHL) strategy to improve living environments and systems based health promotion for Chagas disease (CD) in Ecuador. They find that three factors influence the sustainability of CD disease through HHHL, ‘(1) systemic improvement of families’ quality of life, (2) perceived usefulness of control measures, and (3) flexibility to adapt to emerging dynamics of the context’. They conclude that home improvement as facilitated through a systems-based rather than a disease-specific process enhances population level agency and facilitates community partnerships around CD prevention.

### WASH interventions

WASH interventions are vital for the prevention and control of NTDs, yet they have been under-prioritized in the intervention of global disease control efforts. Waite, Velleman et al [72] identify priority next steps in the collaboration between the WASH and NTD sectors, including ‘building capacity for WASH programming among NTD control teams’, improved ‘coordination at the country level’, and ‘strengthening the epidemiological evidence and operational learning for joint WASH and NTD interventions’. Chard, Levy et al [73] note that despite links ‘between poor access to WASH and risk of enteric diseases, there is mixed evidence of the ability of WASH interventions to mitigate these diseases based on findings from the “gold standard” randomized controlled trials’. Based on a cross-sectional survey of 50 villages in Lao People’s Democratic Republic, they found challenges in addressing enteric infections using many of the existing WASH intervention approaches, including intra-household transmission, animal faeces exposure, and environmental contamination. Another study looking at correlations between WASH interventions and infection was conducted by Tomczyk, Deribe et al [74] however their study also considers personal protective measures namely footwear for NTDs whose route of transmission or occurrence may be through the feet. They found that footwear use was associated with decreased odds of several different NTDs and should be prioritized together with existing NTD interventions to guarantee a reduction of several NTDs and to accelerate their control and elimination. Table 2 summarises the major findings, excluding policy briefs and public statements.

**Table 2:** Summary of major findings, excluding policy briefs and public statements.

Type of Intervention and/or study	NTD	Geographical coverage	Study Design/Method	Aim(s)	Outcome/recommendations
Studies of Knowledge, Attitude, and Practice (KAP)	Human African Trypanosomiasis (HAT)	South Sudan	Cross-sectional KAP survey that utilised questionnaires, key-informant interviews, and a focus group discussion	To (1) elicit communal and individual KAP on HAT and (2) to identify gaps in community KAP and to determine preferred channels and sources of information	90% of respondents had general knowledge on HAT. Myths and stigma are key gaps in community knowledge. The preferred source of communication is the radio.
	Dengue	Malaysia	A school-based pre- and post-tests design whereby a booklet on dengue was distributed during the interphase of the tests to school children where 51.7% were flood victims and the remaining were from unflooded areas	To (1) assess the KAP regarding dengue among school children from flood and unflooded areas and (2) evaluate the effectiveness of the dengue health education program in improving their KAP level	Students from the unflooded area had higher knowledge scores compared to those from the flooded area, while non-significant differences were observed in the attitude and practice between the two study areas. The health education program significantly improved knowledge and practice in the flooded area and knowledge only in the unflooded area.
Assessment of health seeking behaviours	Lymphatic filariasis; podoconiosis; schistosomiasis; soil-transmitted helminths; trachoma	Ethiopia	Interviews and focus group discussions	To explore how gender interacts with NTD service provision and uptake	Gender related factors affected care seeking for NTDs and were described as reasons for not seeking care, delayed care seeking and treating NTDs with natural remedies. Women faced additional challenges in seeking health care due to gender inequalities and power dynamics in their domestic partnerships.
Studies that explore levels of care in health systems	Schistosomiasis	Kenya	Cross-sectional survey	To describe a general contextual analysis that uses multi-level models to partition and quantify variation in individual NTD risk at multiple grouping levels in rural Kenya	Broad-scale contextual drivers shape infectious disease risk in this population, but these effects operate at different grouping-levels for different pathogens. Variation in individual infection risk is partitioned at the household, sublocation and constituency-levels.
	Leprosy	India	Qualitative research using a grounded theory approach	To explore the in-depth contextual attributes of a hospital community that has been able to successfully provide sustainable care for a long time without sustantial external funds	The findings identify the importance of forming a cooperative community and implementing social rehabilitation for sustainable leprosy control. The bottom-up formation of a ‘consumer-provider cooperative’ is particularly successful, where patients mutually support each other with basic treatment learned from experience.
	All NTDs	Tanzania	Qualitative case study design that utilised a mobile phone-based Management Information System (MIS) for the control of NTDs in which village health workers were given mobile phones with web-based software	To test the feasibility of using frontline health workers to capture data at point of source	Providing mobile phones to village health workers helped to increase the efficiency of routine work boosting their motivation and self-esteem. However, the information generated from the mobile phone-based NTD MIS has yet to be used to support decentralised decision-making.The biggest hindrance to local usage of information for health planning is the lack of synthesised and analysed health information from the district and national levels to villages.
	Buruli ulcer	Benin	Intervention-oriented research implemented in four steps: (1) baseline study, (2) training of health district clinical staff, (3) outreach education, (4) outcome and impact assessments	To assess a pilot program designed to introduce buruli ulcer treatment decentralization in a high endemicity district previously served by centralized hospital-based care	71% of all detected buruli ulcer cases could be treated in the community following outreach education, and most of the afflicted were willing to accept decentralized treatment. Community confidence in decentralized care was greatly enhanced by clinic staff who came to be seen as having expertise in the care of most chronic wounds.
	All NTDs	Global	Review of the literature	To review the recent advances and some of the challenges in the fight against NTDs	Strategies that involve mechanisms of ‘Push’ which aims at cutting the cost of research and development for industry and ‘Pull’ which aims at increasing market attractiveness were proposed and verified to be successful. There should be shared responsibility globally to reduce risks, overcome obstacles and maximize benefits. Stakeholders should take concerted and long-term measures to meet multifaceted challenges by alleviating extreme poverty, strengthening social intervention, adapting climate changes, providing effective monitoring and ensuring timely delivery.
	Helminth NTDs	Western Pacific Region	Data summary and literature review	To review the main determinants for helminth NTD endemicity and current control strategies	Multi-sectoral collaboration for helminth NTD control is feasible if the target diseases and sectors are carefully selected. Incentive analysis covering key stakeholders in the sectors is crucial, and the disease-control strategies need to be well understood.
Mass drug administration	Schistosomiasis	Kenya	Longitudinal qualitative study	To explore the experiences, opportunities, challenges as well as recommendations of community health workers (CHWs) after participation in annual MDA activities	Opportunities for implementing MDA include the presence of CHWs, their supervisory structures and their knowledge of intervention areas, and opportunity to integrate MDA with other health interventions. Challenges include lack of incentives, fear of side effects, misconceptions regarding treatment and mistrust, difficulties working in unsanitary environmental conditions, insecurity, and insufficient time.
	Schistosomiasis	Global	Age-structured deterministic model of parasite transmission and control by praziquantel treatment developed by Imperial College London to follow through the currently recommended WHO guidelines for S. mansoni	Using three different age-intensity profiles of infection for Schistosoma mansoni with low, moderate and high burdens of infection in adults to investigate how the age distribution of infection impacts the mathematical model generated recommendations of the preventive chemotherapy coverage levels required to achieve the WHO goals	The optimal treatment strategy for a defined region requires consideration of the burden of infection in adults as it cannot be based solely on the prevalence of infection in school age children.
	NTDs requiring MDA	Uganda	Observation of the similarity/diversity between community-based MDA to predict division of labour and overall village treatment rates	To explore what constitutes the best combination of community medicine distributors (CMDs) for achieving high (>65%/75%) treatment rates within a village	An equitable distribution of labour between CMDs may be essential for achieving treatment targets of 65%/75% within community-based MDA. To improve the effectiveness of CMDs, national programmes should explore interventions that seek to facilitate communication, friendship, and equal partnership between CMDs.
	Schistosomiasis; soil transmitted helminths; trachoma	Togo	Prevalence survey to plan mass drug administration	To conduct a nationwide integrated NTD prevalence survey to define the need for public health interventions using an innovative mapping protocol	The district prevalence of schistosomiasis ranged from 2 to 49% and of soil transmitted helminths from 5 to 70%, with prevalence at the village level ranging from 0 to 100% for both diseases. Two districts passed the threshold of 10% for active trachoma, but the cluster survey indicated this was because of misclassification bias and that the real prevalence was <1%. Results were used by the Ministry of Health and partners to plan integrated MDA.
Disease mapping and modelling	Schistosomiasis	Global	Precision mapping	To understand the impact of precision mapping in schistosomiasis elimination	Precision mapping of schistosomiasis gives high-resolution information at the local level. By increasing map granularity and spatial resolution, precision mapping provides the best evidence-based data to guide interventions in transmission zones and allows for a better and rational utilization of praziquantel and available resources.
	Dengue	Global	Agents-based models (ABM)	To provide an overview of important characteristics of ABM for decision-analytic modelling of infectious diseases. A case study of dengue epidemics illustrates model characteristics, conceptualization, calibration and model analysis.	ABM is a dynamic, individual-level modeling approach that is capable to reproduce direct and indirect effects of interventions for infectious diseases. The ability to replicate emerging behavior and to include human behavior or the behavior of other agents is a distinguishing modeling characteristic.
	All NTDs	Ethiopia	Literature review, including integrated mapping	To underscore the burden of NTDs in Ethiopia, highlight the state of current interventions, and suggest ways forward.	Ethiopia bears a significant burden of NTDs compared to other Sub-Saharan African countries. To achieve success in integrated control of NTDs, integrated mapping, rapid scale up of interventions and operational research into co-implementation of intervention packages is crucial.
	Human African Trypanosomiasis (HAT)	Democratic Republic of Congo	Mathematical model of disease dynamics	To assess the potential impact of changing the intervention strategy in two high-endemicity health zones	It is recommended that a two-pronged strategy including both enhanced active screening and tsetse control is implemented in this region and in other persistent HAT foci to ensure the success of the control programme and meet the 2030 elimination goal for HAT.
	Lymphatic filariasis; trachoma; schistosomiasis; soil-transmitted helminths	Global with field testing in Mali and Senegal	Innovative integrated NTD mapping protocol (Integrated Threshold Mapping (ITM) Methodology)	To develop and test ITM methodology in geographic locations where there is overlap of NTDs.	Each NTD program has its own mapping guidelines to collect missing data. Where geographic overlap among NTDs exists, an integrated mapping approach could result in significant resource savings.
Diagnostic screening and surveillance	Leprosy	The People’s Republic of China	Diagnosis of leprosy using three cardinal clinical signs	To analyse cases of leprosy diagnosis in a post-elimination era and to explore associated factors of grade 2 disability.	Comprehensive case-finding measures including health promotion, personnel training, reward-offering, with an emphasis on former high or middle endemic areas, are necessary to improve early presentation of suspected cases and to increase suspicion and encourage participation of all relevant medical staff. Symptom surveillance based on a powerful transfer center may play an important role in the early detection of new cases in post-elimination era.
	Helminth NTDs	Global	Literature review	To explore helminth NTD control and elimination efforts.	The drugs used to control helminths are not always effective against all life-cycle stages of the parasites. This means that any efforts to control the spread of helminth NTDs will only be effective and sustainable if they are maintained long-term. Considerable progress has been made on the diagnostics and control of neglected tropical helminth diseases. However, further actions are needed to preserve the achievements of current investments in NTD control.
Health economics studies	Schistosomiasis; lymphatic filariasis; soil transmitted helminths	Madagascar	Extended cost-effectiveness analysis (ECEA) methods	To assess the (1) health gains, (2) household financial gains, and (3) education gains for five NTD interventions that the government of Madagascar aims to roll out nationally.	The estimated incremental cost-effectiveness for the roll-out of preventive chemotherapy for all NTDs jointly was USD125 per DALY averted. Per dollar spent, schistosomiasis preventive chemotherapy, in particular, could avert a large number of infections, DALYs, and cases of school absenteeism.
Behaviour change interventions	Trachoma	Global	Review of behaviour change intervention literature	To understand the processes that ensure effective program design, to identify types of interventions, and to understand which programme components should be used.	We must develop interventions, in partnership with communities and local actors, based on the consideration of the behavioural targets, context, and implementation constraints and opportunities.
	Chagas disease	Ecuador	Cross sectional behaviour change study	To determine predictors for intention to prevent home infestation based on the Health Belief Model (HBM).	The gap between behavioral intention and actual infestation reveals the need to assess home practices and their actual efficacy to fully enact and apply the HBM.
	Leishmaniasis	Brazil	Computer game intervention	To inform and encourage changes in behaviours and attitudes in local populations, while involving multidisciplinary teams of healthcare professionals and researchers.	Researchers highlighted the complexity of designing games for the purpose of learning or behaviour change, recommending that designers involve experts from various relevant fields in the process of game design, and pointing out the need for iterative evaluation of the instructional elements, gameplay, and interface components of serious games.
Community engagement, health promotion, health education and WASH intervention studies	Soil transmitted helminths	Philippines	Qualitative cross-sectional study	To analyse the strengths, weaknesses, opportunities, and threats for three intervention components for STH control: mass drug administration (MDA), health education, and sanitation.	School teachers and staff generally perceived MDA to be a well-delivered program, but opportunities exist to strengthen other control strategies: health education and school rules on hygiene and sanitation at school.
	Chagas disease	Ecuador	Qualitative study using the Healthy Homes for Healthy Living (HHHL) health promotion strategy	To explore social factors that contribute, or limit sustainability of Chagas disease’s prevention models focused on home improvement.	Sustainability of the Chagas disease prevention model proposed by HHHL is enhanced by the confluence of three factors: systemic improvement of families’ quality of life, perceived usefulness of control measures, and flexibility to adapt to emerging dynamics of the context.
	Lymphatic Filariasis	Zambia	Exploratory qualitative case study approach using focus group discussions, in-depth and key informant interviews	To explore how community engagement approaches used in MDA for Lymphatic Filariasis shape participation in the programme, with a view of proposing effective engagement strategies.	Facilitating participation in MDA for Lymphatic Filariasis requires designing and implementing effective community engagement strategies that take into account local context, but also seek to explore all avenues of maximizing participation for improved coverage levels.
	Schistosomiasis	Ethiopia	Survey	To present an overview of the Enhanced School Health Initiative (ESHI), which integrates three complimentary health interventions: deworming; school feeding; and provision of a WASH package in schools.	The strategy adopted by the Ethiopian government is leading the way in multidisciplinary, multisectoral programming, providing a model that can be adapted and adopted by other countries.
	Schistosomiasis	Global	Literature review	To (1) compare the different treatment delivery methods based both on coverage of school-aged children overall and on coverage specifically of non-enrolled children and (2) to identify qualitative community or programmatic factors associated with high or low coverage rates.	A combined community and school-based delivery system should maximize coverage for both in- and out-of-school children, especially when combined with interventions such as snacks for treated children, educational campaigns, incentives for drug distributors, and active inclusion of marginalized groups.
	Schistosomiasis	Zanzibar	Qualitative study using interviews	To (1) explore the involvement of Madrassa teachers for behaviour change interventions and (2) to investigate the impact of multiple approaches to eliminate urogenital schistosomiasis transmission from Zanzibar.	Madrassa teachers are influential in the Zanzibari society, and hence are important change agents within our community-level behavioural intervention. They might constitute an untapped resource that can help to expand and increase acceptance of and participation in schistosomiasis and other neglected tropical disease control activities in African Muslim communities.
	Enteric pathogens (Shigella, Salmonella, E. coli) *identified as NTDs by PLOS NTDs, *but not the WHO*	Lao People’s Demogratic Republic	Cross sectional survey	To (1) estimate the prevalence of enteropathogens among children <5, school-aged children, and adults; (2) model associations between WASH transmission pathways and enteropathogen infections; and (3) quantify clustering of enteropathogen infections at the household- and village-level.	WASH access as currently defined does not reveal a measurably protective association with infection for many etiologies. Household- and community-level factors beyond WASH access, such as intra-household pathogen transmission, exposure to animal feces, and contextual factors in the public domain may be important risk factors for enteric infections.
	NTDs for which the route of transmission or occurrence may be through the feet	Global	Literature review	To assess whether footwear use was associated with a lower risk of selected NTDs.	Footwear use was associated with a lower odd of several different NTDs. Access to footwear should be prioritized alongside existing NTD interventions to ensure a lasting reduction of multiple NTDs and to accelerate their control and elimination.
	All NTDs	Global	Literature review	To review progress made in recent years, explores mechanisms supporting advances, and identifies priorities and next steps for accelerating WASH integration.	In order to accelerate WASH integration in NTD control through strong collaborations with the WASH sector, the NTD sector could make use of strong data management skills and advocacy opportunities.
	Schistosomiasis	Tanzania	Mixed methods pilot study to implement an Enhanced Development Governance (EDG)	To explore community participation as an effective strategy for developing sustainable village health governance.	Community participation plays an important role in sustaining improved health outcome. The EDG model is associated with a statistically significant but small decrease in schistosomiasis and diarrhoea. The study provides an innovative way to financially support community health workers.

## Discussion

The two guiding questions this paper aims to address are, ‘what are the approaches and methodologies used in NTD interventions?’ and ‘what characteristics and approaches are used in community health promotion theory that could be applied to NTD interventions?’, while the results section addresses the first question the following discussion section will address the second question by analysing the relationship between NTD interventions and health promotion and will provide recommendations for best practice.

### What can we learn from health promotion?

A participatory and assets-based approach is effective in community level health promotion. However, individual interventions are not effective and need to be considered as part of a bigger programme. For an intervention to be successful it is vital that it be part of a multi-sectorial and multi-level programme; this could be described as a programmatic approach to health promotion [75]. A larger programme includes a collection of interventions that are individually intended to bring about multiple changes in a variety of targets, but which share an overarching aim. Single, one-off interventions are only effective and lasting when they inform a larger programme.

Within the programmatic approach the community can, and should, have a role in individual interventions. There is evidence that community involvement aids successful health promotion, but ‘community’ has a wide range of meanings and should be appropriately defined.

An ecological approach to health includes the developmental history of the individual, psychological characteristics, interpersonal relationships, neighbourhood, organisations, community, public policy, physical environment, and culture [63]. A more recent emphasis on this approach follows the development of the concept of health promotion since the 1980s in WHO policies [76]. Health promotion has shifted away from a focus on the modification of individual risk factors and behaviours to address the context and meaning of health. This is reflected in the WHOs Health for All Strategy where governments are responsible for the health of their people, not just for providing health services; those services must do something. This strategy shifts away from disease prevention and towards capacity building for health, which in the NTD literature would suggest WASH campaigns and universal healthcare. NTD interventions illuminate the importance of complementary prevention strategies alongside direct interventions like MDA. Thus, the WHOs Health for All Strategy ought to incorporate capacity building and other preventative measures when it comes to addressing NTDs.

Capacity building not only serves to support prevention measures, but when coupled with an ecological model they can work together to tackle social inequalities in health. Whitehead [77] has identified a typology of actions to reduce health inequalities, many based on capacity building and system strengthening. She suggests strengthening individuals and communities, improving living and working conditions, and promoting healthy macro-policies. She critiques interventions that seek to strengthen individuals that are not programmatic in nature. For example, educational campaigns rarely work in isolation and are particularly ineffective for disadvantaged populations and areas. While health education remains a component of health promotion, there has been movement away from individually focused health education interventions [78] Tilford suggests that interventions should be participatory in nature where individuals and communities have active participation in health promotion interventions. Health education and educational campaigns must be combined with other initiatives that address structural barriers to meeting education goals [77].

The need to consider ecological and structural barriers in health promotion interventions is evident in NTD interventions as well, for example with the need to identify environmental determinants associated with helminth NTDs. Whitehead further argues that interventions that focus on individual and community strengthening have primarily focused on deprived groups and have not involved wider sections of society. However, there is evidence that disparities in health may be exacerbated by population level interventions[79]. Yet, the decision for a health intervention to target micro or macro levels must also account for epidemiological disease considerations. For example, in the ‘high risk’ approach susceptible individuals are protected, while in the population approach the causes of incidence are controlled [80]. These two approaches are not necessarily in competition, but from an epidemiological point of view there is emphasis on discovering and controlling the causes of incidence. This suggests that NTD interventions must balance controlling causes of incidence along with protecting the vulnerable, and not exacerbate health inequality along the way.

Additionally, interventions tend to focus on treating symptoms rather than underlying causes of the problem [77]. This focus on not only treating symptoms but also addressing the underlying causes of disease is part of an approach called ‘salutogensis’ [81]. Salutogensis is an approach that focuses on factors that support human health and well-being rather than on factors that cause disease. It is by identifying and acting on underlying causes of disease, like access to clean water in the case of many NTDs, that a theory of change can be developed. Theories of change include collective action through community participation while drawing on and building capacity within existing social capital; this is part of an assets approach to health promotion.

Participation in health promotion must involve an integration of social norms perspectives if it intends to be truly participatory. Beniamino and Lori [82] identify several key points if social norms are to be successfully incorporated: social norms and attitudes can differ and they can coincide; protective norms can provide resources of achieving social improvement in health related practice; harmful practices need to be understood within their web of interactions; the prevalence of a norm is not necessarily indicative of its strength; norms can both direct and indirect influence; publicising the prevalence of a harmful practice can exacerbate it; and people-led social norm change is ‘the right’ and ‘the smart’ approach.

### Framework for best practice- NTD interventions and health promotion

**Fig 2:**
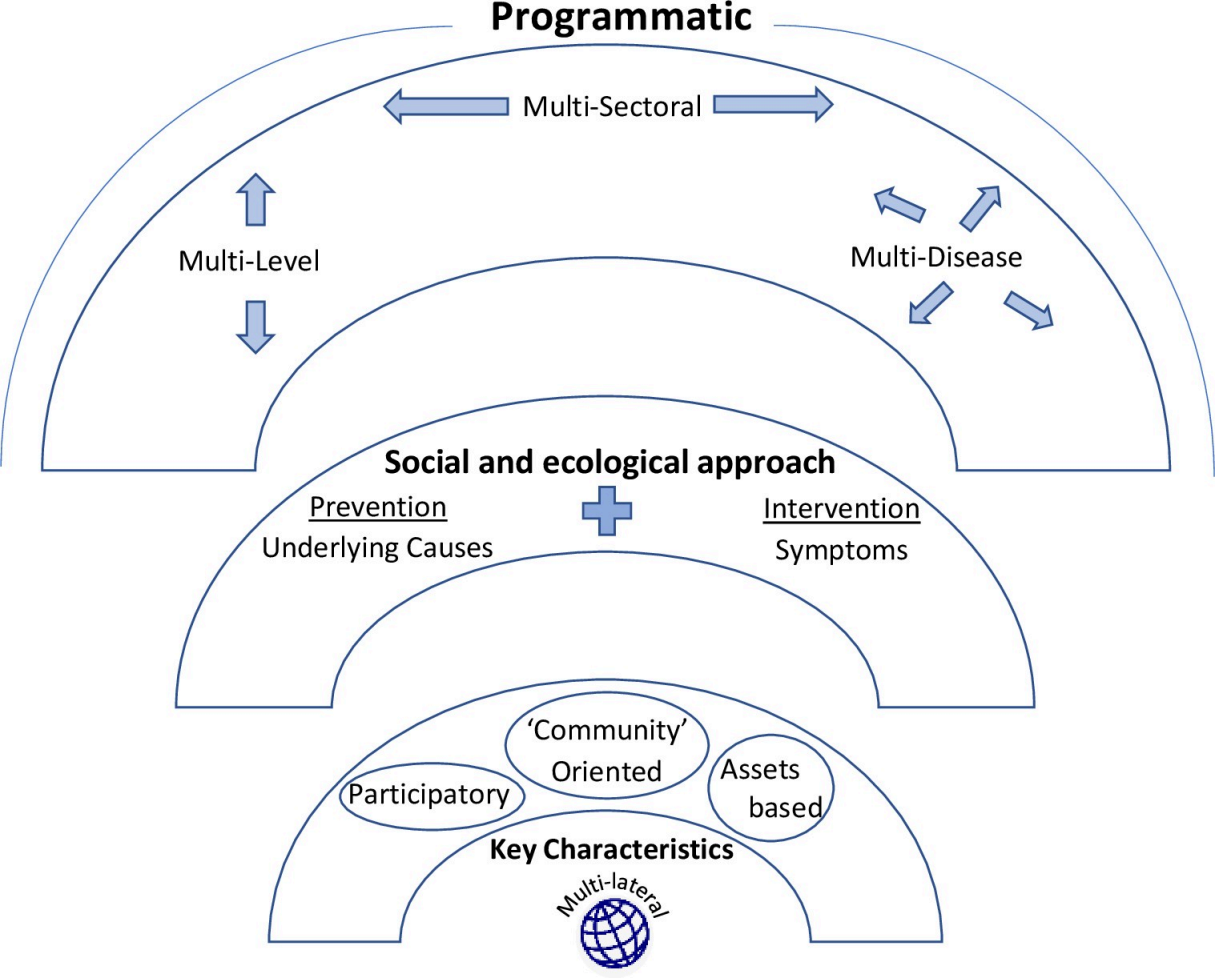
Framework for best practice.

There are many lessons to be learned from NTD interventions and health promotion theory. There are points overlapping between good practice in NTD interventions and health promotion theory, and there are treatment-specific aspects of NTDs that should be considered even if they might not be considered best practice in health promotion theory. Based on the literature, we have identified the four core components of best practices including programmatic interventions, multi sectoral and multi-level interventions, adopting a social and ecological model and clearly defining ‘community’. The following figure (Fig 2) shows the framework for best practice.

In the literature on NTD interventions it is apparent that MDA is vital to treat the symptoms of many NTDs. However, health promotion theory emphasises treating the underlying causes of disease, something that the literature on NTD interventions has acknowledged through the call for WASH activities and universal health care. Additionally, there have been many examples of MDA being rejected in communities. Hastings [83] writes about an MDA intervention for urinary schistosomiasis and soil-transmitted helminths in Tanzania, in which villagers protested and accused her of bringing medicine to poison the children. She highlights the differences between biomedical and local understandings of these diseases, while calling for a ‘biosocial’ approach to MDA programmes. Such an approach considers the local social, biological, historical, economic, and political contexts that programmes are implemented.

Parker, et al [84] also write about programs to control schistosomiasis and soil-transmitted helminths, but in Uganda. They also show that adults reject free treatment due to a divergence between biomedical and local understandings of the disease and note there is inappropriate and inadequate health education on this treatment. They call for more effective delivery strategies and a more socially appropriate approach to behavioural change, including consideration of the social, economic, and political aspects of drug distribution.

The framework below (Fig 2) takes best practice from NTD interventions and health promotion theory to provide guidance in developing future NTD interventions. First, interventions must be programmatic in nature, meaning that a one-off intervention will be ineffective if they are not part of a larger and systematic approach to treating NTDs. This programmatic approach ought to be multi-sectoral, multi-level, address more than one disease, and when possible be multi-lateral. Interventions should involve governmental departments and organisations working across sectors to have the most robust approach. For example, a Ministry of Health must work in collaboration with other ministries that protect vulnerable groups (e.g. women, youth, and children) or protect basic resources and essentials for survival (e.g. environmental affairs and agriculture). Additionally, there might be efforts across departments there are unexpected. It should be noted that this should not create competition for resources, rather it should foster greater transparency and collaboration. Interventions should also be multi-level and follow the ecological model of levels of influence on behaviour to include the individual, interpersonal, organisational, community, and policy levels.

A social and ecological model is important to adopt when developing and implementing NTD interventions. It includes addressing not only the symptoms of disease through activities like MDA, but also the underlying causes of disease. In this model, underlying causes include environmental determinants, the developmental history of individuals, psychological and social influences, and public policy. Each of these underlying causes should be addressed in a programmatic approach.

Several key characteristics ought to be included in NTD interventions including that it is participatory in nature, ‘community’ oriented, and assets based. It is important the ‘community’ is clearly defined in each intervention, and that community members are included in intervention activities and viewed as assets to interventions. Community norms and behaviours, and activities should be incorporated and not viewed as barriers to interventions.

Finally, several overlooked factors should be incorporated into a framework of best practice, namely capacity building in disease endemic countries (DECs) and gendered components of NTDs. PLOS Neglected Tropical Diseases acknowledges that it is important to promote and profile the efforts of scientists in DECs to build strong science and health capacity while providing open access to essential information and a platform for publication [85]. There is also little discussion on the influence of gender in NTD programs and interventions despite known gender specific differences in disease prevalence, transmission, and exposure. Wharton-Smith, Rassi et al [86] conducted a qualitative study in Ethiopia to explore just this and found that ‘gender as it related to health care seeking was generally discussed along two themes: (1) shame and fear of disclosing certain NTD symptoms and (2) power dynamics’. There ought to be more research into shame, fear, and sexual and social taboo along with power dynamics in domestic partnerships to better understand the reasons women face in not seeking care, delayed care seeking, and treating NTDs with natural remedies.

### Limitations

We identify several potential limitations of this scoping review study. We acknowledge that scoping studies ‘map’ relevant literature in the field(s) of interest [16] and might therefore be less robust than systematic reviews due to their broader scope and a lighter quality assessment [87,88]. However, scoping reviews still offer a high level of research [87,89]. We also acknowledge the potential for bias during the selection of studies for inclusion and the synthesis of studies [89]. Only studies published in English and from 2000 were included in this scoping review, thus introducing an element of inclusion bias. To address potential bias when synthesising studies the authors appraised the quality of studies using the CASP qualitative checklist and consensus was reached through discussion.

## Conclusion

What is evident from the literature is that NTD interventions tend to centre on MDA, which focuses on both prevention and treatment. In MDA entire communities are given drugs- healthy people as well as the unhealthy. This suggests a need for an approach that includes MDA along with multiple strategies that inform a larger multi-disease and multi-sectoral programme. One gap the literature illuminates is the need to apply this approach to policy as well. Current strategies include a focus on WASH and need to incorporate the social and ecological determinants of NTDs–often WASH related. WASH is deemed a vital component for any NTD intervention, so much so that the WHO specifically developed a toolkit to accompany NTD interventions [90]. WASH includes education and campaigns, as well as infrastructural development like improving access to safe water. This suggests a preventative and systems approach to health, not just a treatment-based approach.

The literature also indicates that a long term and systematic approach to the social and ecological determinants of NTDs is an effective prevention measure [37,38]. Developing strong communities and incorporating social rehabilitation at the sublocation level (e.g. hospital) could benefit several NTDs and infectious diseases through a multi-disease, multi-sectoral, and multi-lateral [39,40] approach. Additionally, the flow of information needs to go beyond case reporting at the village level upwards, but also health planning at the district and national levels needs to flow down to village health workers at the frontline of NTD care [42]. Several studies suggest that targeting NTDs through holistic care with a two-way flow of information could go beyond immediate health impacts by contributing to health systems, sustainable development, raising educational attainment, increasing productivity, and reducing health inequalities.

## Supporting information

S1 Appendix Electronic databases. (DOCX)
