# Supplementary material for: Scoping review of Neglected Tropical Disease Interventions and Health Promotion: A framework for successful NTD interventions as evidenced by the literature
Source: PLoS Negl Trop Dis. 2021 Jul 6;15(7):e0009278. doi: 10.1371/journal.pntd.0009278 (PMC8321407; doi:10.1371/journal.pntd.0009278)
Supplement: S1 Appendix — (DOCX) [file pntd.0009278.s001.docx]

S1 Appendix. Electronic databases

| Database/platform | SciVerse Scopus (Elsevier) |
| --- | --- |
| Date coverage: | 2000-2020 |
| Library: | Access through University of Sussex |
| Date of search: | 15/5/2020 |
| Limits: | 1. Research articles   [Control]Book chapters  2.  Case reports  3.  Data articles  4.  Discussion  5.  Editorials  6.  Practice guidelines |
| Search query: | ((ntd OR neglected tropical disease) AND (intervention) AND (community) AND (health promotion)) |
| Number of hits: | 513 |
| Articles after relevance screening: | 11 |
| Articles after data characterization with CASP and manual removal of duplicates: | 10 |

| Database/platform | MEDLINE (PubMed) |
| --- | --- |
| Date coverage: | Full text coverage |
| Library: | Free access |
| Date of search: | 15/5/2020 |
| Limits: | none |
| Search query: | (("ntd") OR "neglected tropical disease") AND "intervention" |
| Number of hits: | 215 |
| Articles after relevance screening: | 19 |
| Articles after data characterization with CASP and manual removal of duplicates: | 16 |

| Database/platform | Academic Search Complete: EBSCO |
| --- | --- |
| Date coverage: | 2000-2020 |
| Library: | Senate House, University of London |
| Date of search: | 20/5/2020 |
| Limits: | English articles only |
| Search query: | neglected tropical diseases AND ((interventions or strategies or best practices) OR (health promotion)) |
| Number of hits: | 865 |
| Articles after relevance screening: | 44 |
| Articles after data characterization with CASP and manual removal of duplicates: | 40 |
